# Exploring cutoff points and measurement invariance of the Brunnsviken brief quality of life inventory

**DOI:** 10.3389/fpsyg.2023.1305682

**Published:** 2024-01-08

**Authors:** Jón Ingi Hlynsson, Philip Lindner, Bushra Barri, Per Carlbring

**Affiliations:** ^1^Department of Psychology, University of Iceland, Reykjavík, Iceland; ^2^Centre for Psychiatry Research, Department of Clinical Neuroscience, Karolinska Institutet and Stockholm Health Care Services, Stockholm, Sweden; ^3^Department of Psychology, Stockholm University, Stockholm, Sweden

**Keywords:** Brunnsviken brief quality of life inventory, measurement invariance, psychometric evaluation, generalized anxiety disorder questionnaire, patient health questionnaire

## Abstract

**Introduction:**

Quality of life (QoL) can be defined as the goodness of life, beyond simply absence of disease or functional impairments, self-rating scales of which capture valuable information beyond change in primary outcomes. This study (*n* = 3,384) validated the Brunnsviken Brief Quality of Life Inventory (BBQ) across divergent groups by evaluating its measurement invariance (MI). We hypothesized measurement invariance for the BBQ across age groups, genders, depression, and anxiety severity. Potential cutoff points for the BBQ were also explored.

**Method:**

Confirmatory factor analysis (CFA) models were fit to sample data obtained from an ongoing study on transdiagnostic internet-based treatment modules. Parameters were successively constrained to assess configural, metric, scalar, and residual invariance factor structures across different groups.

**Results:**

The BBQ demonstrated MI at the metric level and partial MI at the scalar level across all these groups, which remained stable at the strict-residual level for all groups except for genders. These results remained stable after correcting for unbalanced group sizes for gender, clinical–subclinical levels of depression, and clinical–subclinical levels of anxiety. A cutoff point analysis revealed that a BBQ total scores below 39 was associated with notable psychopathology.

**Discussion:**

The BBQ is a reliable measure of QoL that is applicable for various divergent groups (e.g., vulnerable persons), and thus a viable instrument for use in healthcare and research with minimal aversive impact.

**Clinical trial registration**: NCT05016843.

## 1 Introduction

Studies on negative emotions and the trajectory of negative emotional experiences are plentiful within the field of psychology (Lahey, 2009; Sauer-Zavala and Barlow, 2021). Such investigations have, for instance, produced robust data on what factors predispose individuals for emotional disorders (Sauer-Zavala and Barlow, 2021). However, positive aspects such quality of life (QoL) have historically been conceptualized with much less rigor. First conceptualized in the early 1960s, the concept of QoL has evolved substantially over time. Initially, QoL was defined as the absence of negative health conditions (e.g., the “five D’s”: death, disease, disability, discomfort, and dissatisfaction), but has increasingly been formulated as a more positive valanced construct (Pennacchini et al., 2011). For instance, according to the World Health Organization (2014), QoL encompass how an individual perceives their physical, mental, and social well-being within their cultural context, while considering their goals, expectations, and concerns (WHOQOL Group, 1995). Hence, QoL is viewed as a subjective and multi-faceted construct, extending beyond the mere absence of unfavorable life conditions. Furthermore, although psychopathology can be expected to correlate with lower QoL, these are not isomorphic constructs and should thus be measured independently of each other (Frisch, 1998). Indeed, indicators of QoL can provide valuable information about psychotherapeutic treatment efficacy and effectiveness for researchers and practicing clinicians that is not captured by scales measuring the absence of various pathological symptoms (i.e., both psychopathological such as anxiety and depression as well as physiopathology such as diseases). Nevertheless, there is little consensus within the wider research community regarding a common operational definition or definitive theoretical framework for defining QoL; although it can be defined in general terms as the goodness of life (Bowling, 2005).

In order to understand QoL as a global marker of goodness, it is useful to conceptualize it in both a micro and macro terms (Bowling, 2005). The former places emphasis on the subjective components associated with QoL (e.g., perceived life satisfaction, expectations, optimism, uncertainty; Allison et al., 1997), while the latter encompasses more objective life components (e.g., income, housing, education, temperament; Spiro and Bossé, 2000; Bowling, 2005). The components and constituent parts of these subjective and objective domains are additive factors that interact with one another (*cf.* Maslow, 1943), resulting in a global wellness factor–or marker of the goodness of life. The intricate causal web of associations nested in QoL, however, has led some researchers to conceptualize QoL multidimensionally (see Beckie and Hayduk, 1997). However, although QoL can be intuitively understood as a multidimensional construct with multiple interacting causal components, the number of causes does not necessarily define its dimensionality. It is completely valid to assert that QoL can be dynamically influenced by multiple factors at once, while still being considered a unidimensional construct (Beckie and Hayduk, 1997).

Numerous valid quality of life instruments have been developed to reliably measure QoL (Berzon et al., 1995). However, many are emphasize the absence of symptoms (e.g., the Sheehan Disability Scale [Sheehan et al., 1996], the RAND-36 [Hays et al., 1993]), making them arguably inappropriate for use in psychological research. Other measures such as WHOQOL (WHOQOL Group, 1995) and WHOQOL-BREF (WHOQOL Group, 1994) do not uniformly emphasize the absence of symptoms directly, but are constrained by their multifaceted nature with a focus on symptomological correlates through items assessing sleep disturbances, amount of medical treatment needed to function in daily life, acceptance of one’s bodily appearance, and frequency of anxious and depressed mood (see, e.g., item F1.2 in WHOQOL: “Do you worry about your pain or discomfort?”; WHOQOL Group, 1995). Other established QoL inventories, such as the Quality of Life Inventory (Frisch et al., 1992) and Quality of Life Enjoyment and Satisfaction Questionnaire (Endicott et al., 1993), are constrained by inaccessibility.

Although numerous scales corresponding to subjective well-being (for a review, see Diener et al., 2017) are accessible in various languages, QoL differs from subjective well-being in several ways. Firstly, subjective well-being is generally considered a multidimensional construct, consisting of at least an affective and a cognitive-judgemental component, which limits its interpretability in clinical practice. That is, unidimensional measurements that are conceptually redundant, as opposed to grammatically redundant, ease the interpretability of test scores (Cortina et al., 2020). For instance, the Satisfaction with Life Scale (Diener et al., 1985) consists of six items that can be considered crude indicators of subjective well-being (Diener et al., 2017), and is thus arguably not conceptually redundant. Moreover, some have argued that the literature on subjective well-being has overemphasized the cognitive-judgemental component of subjective well-being (Kjell et al., 2016) which has lead to the creation of scales to supplement measurements of subjective well-being (e.g., tapping into cognitive well-being). However, as noted above, QoL ought to be construed as a global wellness factor that combines affective and cognitive-judgmental evaluations of one’s evaluation of the goodness of life.

The Brunnsviken Brief Quality of life scale (BBQ; Lindner et al., 2016), which was specifically designed to address these limitations, is a brief and easily accessible self-report questionnaire, covering life domains empirically shown to be linked to subjective life satisfaction. Previous studies have shown the BBQ to be a valid QoL measurement (Lindner et al., 2016; Biliunaite et al., 2021; Pantić et al., 2023). However, the operational characteristics of the BBQ across age groups, genders, and psychopathology (e.g., clinically depressed or sub-clinically depressed) has yet to be investigated. The current study aims to address this gap in the literature by examining whether the BBQ possesses measurement invariance (MI) across multiple differing groups. Measurement invariance ensures that the scale measures the same construct consistently across different groups, allowing for meaningful comparisons. To that aim, the following hypotheses were formulated: (1) The BBQ is measurement invariant across age groups, (2) genders, (3) levels of depressive symptom severity, and (4) levels of anxiety symptom severity. Additionally, this study aims to suggest a preliminary set of cutoff points for QoL to enhance the practical application of the BBQ.

## 2 Method

### 2.1 Participants and recruitment

Data for the current study came from an ongoing trial of internet-delivered, transdiagnostic treatments for anxiety and depression (ClinicalTrials.gov identifier: NCT05016843), conducted in Sweden. Participants (*n* = 3,401) were recruited online through a website (Vlaescu et al., 2016) outlining the study’s aims and constituent parts. The study was advertised on Facebook but also spread through word of mouth. As such, the present study consists of treatment-seeking individuals, encompasses a mix of subclinical individuals, clinical participants, including those presenting with severe psychopathological problems, who were part of the analysis but later excluded from the larger treatment study. Thus no strict inclusion or exclusion criteria were set for this study; including all participants allowed us to circumvent a potential restriction of range and increase the power of our statistical analyses.

#### 2.1.1 Sample characteristics

In total, the study included 3,384 treatment-seeking participants, of which 2,477 (73%) were included in the subsequent clinical trial. See Table 1 for descriptive sample characteristic statistics for the present study.

**Table 1:** Descriptive statistics and sample characteristics.^1^

Age	42.44 (12.58) [18–86]
*Gender*	
Female	2,791 (82.5%)
Male	568 (17.8%)
Other gender identity	25 (0.7%)
*Education*	
Elementary school	164 (4.8%)
High school	987 (29.2%)
College level education ≤3 years	865 (25.6%)
College level education >3 years	1,368 (40.4%)
*My socioeconomic status is…*	
much worse than others’	235 (7.9%)
worse than others’	793 (23.4%)
about the same as others’	1,468 (43.4%)
better than others’	796 (23.5%)
much better than others’	92 (2.7%)
*Living with children under 18*	
No	2,000 (59.1%)
Yes	1,317 (38.9%)
Complicated/Sometimes	67 (2.0%)
Usage of pharmaceuticals for depression or anxiety	1,029 (30.4%)
*Occupation*	
Working	2,226 (65.8%)
Studying	451 (13.3%)
Seeking work	233 (6.9%)
Retired	156 (4.6%)
Parental leave	43 (1.3%)
Sick leave	275 (8.1%)
BBQ: total score	36.1 (18.1) [0–96]
PHQ-9: total score	12.8 (5.5) [0.0–27.0]
GAD-7: total score	10.2 (4.9) [0.0–21.0]

Seventeen participants reported being below 18 years of age and were subsequently removed from the analysis.

1 *n* (%); Mean (SD) [Minimum–Maximum].

It should be noted that the sample is overrepresented by females. The reasons for this are undoubtedly multifaceted and a thorough examination of the differential gender representation is beyond the scope of this paper. However, a few key reasons bear mentioning. Firstly, the data originates from a clinical trial that assesses transdiagnostic treatments for anxiety and depression, both of which are more prevalent in females than males (American Psychiatric Association, 2022). Secondly, all participants were treatment-seeking. There is mounting evidence that indicates that trait neuroticism predicts treatment-seeking behavior and that females compared to males have on average higher scores on neuroticism (for a review, see Sauer-Zavala and Barlow, 2021). Finally, there is increasing support for the notion that females more frequently seek out psychotherapy than males (Tedstone Doherty and Kartalova-O’Doherty, 2010; Wendt and Shafer, 2016). Taken together, the overrepresentation of females in our sample may bear some resemblance to the general proclivity to seek out and require psychotherapeutic interventions.

### 2.2 Measures

Demographic variables, anxiety, depression, and QoL measurements were collected during screening.

#### 2.2.1 Brunnsviken brief quality of life inventory

The Brunnsviken Brief Quality of Life Inventory (BBQ) is a freely available, 12-item self-report questionnaire that assesses subjective quality of life across six life areas: Leisure, View on Life, Creativity, Learning, Friends and Friendship, and View on Self (see Lindner et al., 2016 for the full scale). Item-pairs appear sequentially, with an Importance-item for each life area following a Satisfaction-item. Each item is rated on a Likert scale from 0 to 4. The BBQ total score, ranging from 0 to 96, is computed by summing the weighted satisfaction ratings (i.e., by multiplying the Satisfaction and Importance items for each life area and summing the six products for a total score). Item-level data (e.g., used for Cronbach’s alpha calculations and factor analyses) thus correspond to item-pairs (i.e., weighted satisfaction ratings). Recent studies have called into question the equal-weight, equal-importance assumptions that underlies many alternative conceptualizations and measures of QoL (Hsieh, 2022). Thus, the inclusion of importance items and the subsequent usage of weighted satisfaction ratings in the BBQ ought to be construed as a further strength of the validity of the BBQ as a marker of QoL. Previous studies have shown the BBQ to be a unifactorial measure of QoL, with good concurrent and convergent validity, high internal and test–retest reliability, and accurate classification ability in both research and clinical settings (Lindner et al., 2016; Biliunaite et al., 2021; Pantić et al., 2023). In this study, the BBQ demonstrated high internal reliability during screening, Cronbach’s alpha = 0.74 [95% CI: 0.73, 0.75], indicating the BBQ to be a homogeneous scale with good internal consistency.

#### 2.2.2 Patient health questionnaire 9-item scale

The Patient Health Questionnaire 9-item scale (PHQ-9) is a self-report questionnaire that quantifies depressive symptom severity (Kroenke et al., 2001). Each item is rated on a 4-point scale (0–3), with a total score range of 0–27, where a score of 10 or higher is a diagnostic indicator of depression (Kroenke et al., 2001, 2010). The PHQ-9 consistently demonstrates high reliability and discrimination ability in various clinical settings (e.g., internet administration) and the general population (Kroenke et al., 2001, 2010; Kocalevent et al., 2013; Martin-Key et al., 2022). In this study, the PHQ-9 exhibited good internal reliability during screening, Cronbach’s alpha = 0.81 [95% CI: 0.80, 0.82].

#### 2.2.3 Generalized anxiety disorder 7-item scale

The Generalized Anxiety Disorder 7-item scale (GAD-7) is a self-report questionnaire that quantifies anxiety symptom severity (Spitzer et al., 2006; Kroenke et al., 2010). Each item is rated on a 4-point scale (0–3), with a total score range of 0–21, where a score of 8 or higher is a diagnostic indicator of an anxiety disorder (Spitzer et al., 2006). The GAD-7 consistently demonstrates high reliability and discrimination ability in various clinical settings (e.g., internet administration) and the general population (Löwe et al., 2008; Johnson et al., 2019; Byrd-Bredbenner et al., 2021; Martin-Key et al., 2022). In this study, the GAD-7 exhibited good internal reliability during screening, Cronbach’s alpha = 0.84 [95% CI: 0.83, 0.85].

### 2.3 Statistical analyses

Data analysis was conducted using R (R Core Team, 2021). A preliminary analysis included a review of means and item-level correlations. To avoid a restriction of range on the self-report questionnaires, all available screening (i.e., both included and excluded trial participants) were used in the analyses.

To assess whether the factor structure of the BBQ was stable across different groups, measurement invariance (MI) analyses were performed. Confirmatory factor analysis (CFA) models were fit with the *lavaan* package; version 0.6–16 (Rosseel, 2012); using a weighted least squares means and variance adjusted (WLSMV) estimator, which estimates factor loadings with more precision when data are ordinal or categorical (Beauducel and Herzberg, 2006). CFA models were plotted using the *semPlots* package (Epskamp, 2022). Thereafter, parameters were successively constrained to assess configural, metric (loadings), and scalar (loadings and indicator means/intercepts) invariance factor structures across groups. If scalar invariance could not be established, parameters expected to have a significant impact on model fit (i.e., parameters with *p* < 0.05) were freely estimated (i.e., released) in an adjusted model to establish partial scalar invariance. Finally, residual invariance (loadings, indicator means/intercepts, and residuals) was examined where doing so was appropriate (Gregorich, 2006; van de Schoot et al., 2012; Kline, 2023). A poor model fit in any of these models indicates that the constrained parameter differentially operates across groups.

Model fit was evaluated using several fit indices; i.e., the Comparative Fit Index (CFI), Root Mean Square Error of Approximation (RMSEA), and Standardized Root Mean Square Residual (SRMR); as the χ^2^ test statistic is overly sensitive to small and unimportant deviations from an idealistic model fit (Putnick and Bornstein, 2016). Hu and Bentler (1999) noted that although designating specific cutoff values for each fit index is difficult due to them operating differently across various conditions, they suggest that a cutoff value close to 0.95 on the CFI, a cutoff value close to 0.06 for RMSEA, and a cutoff value close to 0.08 for SRMR results in lower Type II error rates (with acceptable costs of Type-I error rates). Building on this, suboptimal model fit was defined as a CFA value above 0.90, SRMR values below 0.10, and RMSEA values between 0.08 and 0.10; adequate model fit was defined as a CFA value between 0.92 and 0.95, SRMR values between 0.08 and 0.10, and a RMSEA value below 0.08; and good model fit was defined as a CFA value between 0.92, SRMR values below 0.08, and RMSEA value below 0.05. Finally, measurement invariance across groups in each model was determined when Δχ^2^ *p* > 0.05, ΔCFI <0.01, ΔRMSEA <0.015 and ΔSRMR <0.030 (for metric invariance) or ΔSRMR <0.015 (for scalar and residual invariance; Putnick and Bornstein, 2016; Bikos, 2022).

Finally, the data was reanalyzed by implementing a subsampling approach to examine MI across groups that were unbalanced in size (i.e., gender and clinical-subclinical levels of anxiety and depression) to substantiate the results. This method involved randomly selecting subsets from the larger group to match the size of the smaller group and conducting invariance testing across 100 replications (Yoon and Lai, 2018).

To provide some preliminary cutoff points for low vs. high QoL according to the BBQ, Receiver Operating Characteristic (ROC) curve analyses were performed using the *pROC* package (Robin et al., 2011). Under the assumption that psychopathology is typically associated with reduced QoL (Hohls et al., 2021; Jenkins et al., 2021), a binary outcome was defined based on the PHQ-9 and GAD-7 scales, with scores of 10 or higher on the PHQ-9 (Kroenke et al., 2001, 2010) and/or 8 or higher on the GAD-7 (Spitzer et al., 2006) were considered indicative of significant psychopathology. Subsequently, a cutoff point was determined as the threshold that maximizes both sensitivity and specificity, essentially representing the point on the ROC curve closest to the coordinate (0.1).

## 3 Results

### 3.1 Confirmatory factor analysis

**Figure 1:**
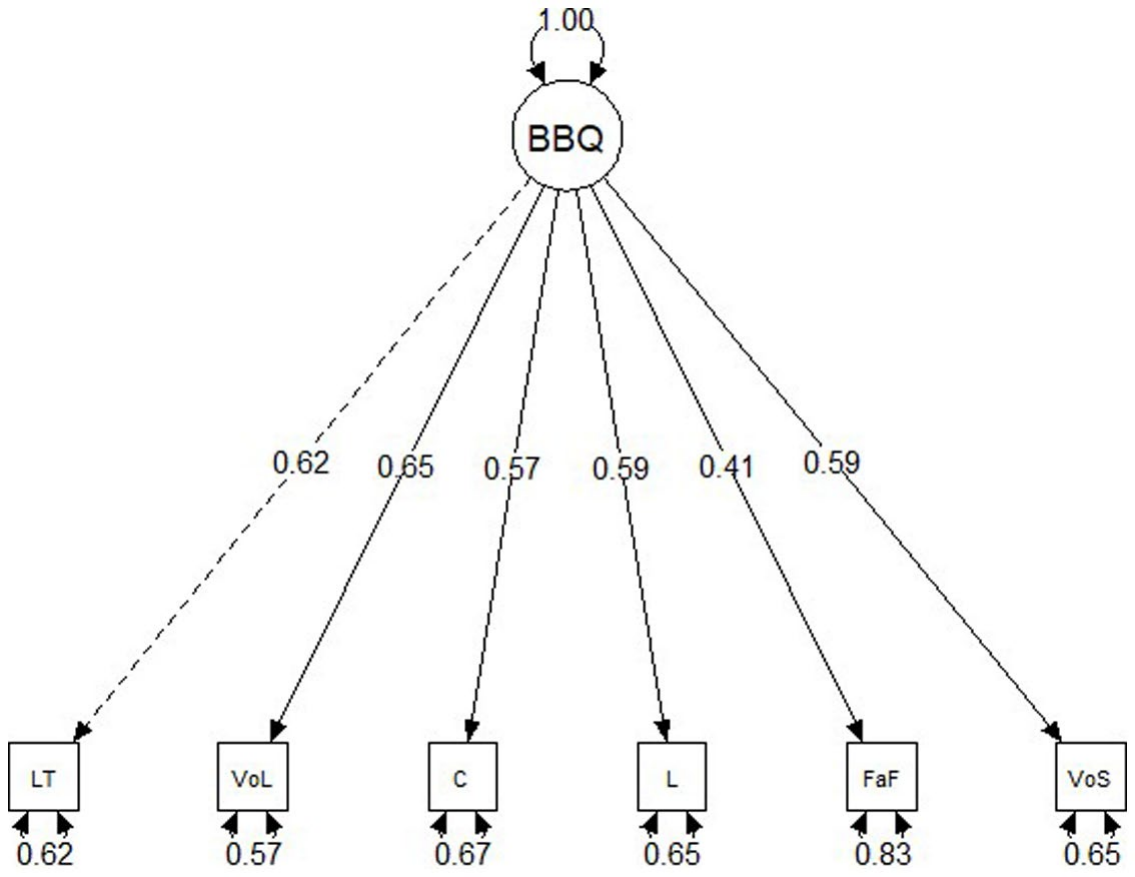
Structural equation model path diagram with standardized factor loadings for the Brunnsviken Brief Quality of Life Inventory (BBQ). LT, Leisure Time; VoL, View on Life; C, Creativity; L, Learning; FaF, Friends and Family; VoS, View of Self.

To assess whether a single-factor solution fit the data, a confirmatory factor analysis was conducted. The BBQ was explicitly developed to match a singular QoL construct and previous studies have provided support for a unidimensional factor solution (Lindner et al., 2016). Thus item-level data (i.e., weighted satisfaction ratings derived by multiplying the Satisfaction and Importance items for each life area and summing the six products for a total score) were fitted to a single factor (see Figure 1).

A single factor solution for the BBQ resulted in a good fit for the data: χ^2^(18) = 146.14, *p* < 0.001, CFA = 0.972, SRMR = 0.049, RMSEA = 0.067 [90% CI: 0.058, 0.087]. Thus we proceeded to assess the measurement invariance (MI) of the BBQ across age groups, gender, levels of depression, and levels of anxiety. See Table 2 for item-level mean scores and correlations on the BBQ.

**Table 2:** Means, standard deviations, and correlations for the BBQ.

					Item		
	*M*	*SD*	1	2	3	4	5
1. Leisure time	0.46	0.27					
2. View on life	0.47	0.27	−0.13				
3. Creativity	0.44	0.29	0.06	−0.21			
4. Learning	0.45	0.28	−0.14	−0.18	0.29		
5. Friends and friendship	0.37	0.31	−0.19	−0.33	−0.54	−0.50	
6. View of self	0.45	0.28	−0.26	0.31	−0.40	−0.24	−0.20

The domains of the BBQ were derived using weighted satisfaction ratings (i.e., by multiplying the satisfaction and importance items for each life area).

### 3.2 Measurement invariance

#### 3.2.1 Measurement invariance of Age

The sample was stratified by the median age of participants (median age = 42) into two groups, above median age (*n* = 1711) and below median age (*n* = 1,673). Participants that reported being below 18 years of age (*n* = 17) were excluded from the analysis. Thereafter, BBQ item-level data were fit to one factor. Fit statistics for all invariance tests are displayed in Table 3. The configural model, which constrained only the relative configuration of item-level data to be equal across the age groups, had an adequate fit to the data: χ^2^(18) = 151.24, *p* < 0.001, CFI = 0.973, SRMR = 0.044, RMSEA = 0.066 [90% CI: 0.057, 0.076]. The metric invariance (weak) model constrained the configuration of item-level data and factor loadings to be constant across the age groups. Fit indices were comparable to the configural model: χ^2^(23) = 160.31, *p* < 0.001, CFI = 0.972, SRMR = 0.046, RMSEA = 0.059 [90% CI: 0.051, 0.068]. Metric invariance was supported by non-significant difference tests that evaluated model similarity: Δχ^2^(5) = 7.58, *p* = 0.18; ΔCFI = −0.001.

**Table 3:** Goodness-of-fit indicators for structural equation modelling analyses for BBQ.

Models/samples	*χ* ^2^	*df*	CFI	SRMR	RMSEA	90% CI
*BBQ age groups*						
Configural model	151.24*	18	0.973	0.044	0.066	[0.057, 0.076]
Metric model	160.31*	23	0.972	0.046	0.059	[0.051, 0.068]
Scalar model	204.91*	28	0.964	0.050	0.061	[0.053, 0.069]
Adjusted scalar model	165.79*	27	0.972	0.046	0.055	[0.047, 0.063]
Strict model	178.66*	33	0.970	0.048	0.051	[0.044, 0.059]
*BBQ gender groups*						
Configural model	141.86*	18	0.974	0.043	0.064	[0.054, 0.074]
Metric model	147.48*	23	0.974	0.044	0.057	[0.048, 0.066]
Scalar model	198.01*	28	0.965	0.049	0.060	[0.052, 0.068]
Adjusted scalar model	151.25*	24	0.974	0.044	0.056	[0.048, 0.065]
Strict model	164.65*	30	0.972	0.047	0.052	[0.044, 0.059]
*BBQ depression groups*						
Configural model	165.78*	18	0.962	0.046	0.070	[0.060, 0.080]
Metric model	175.97*	23	0.961	0.048	0.063	[0.054, 0.072]
Scalar model	232.21*	28	0.948	0.054	0.066	[0.058, 0.074]
Adjusted scalar model	178.60*	25	0.961	0.048	0.060	[0.052, 0.069]
Strict model	182.32*	31	0.962	0.049	0.054	[0.046, 0.061]
*BBQ anxiety groups*						
Configural model	161.25*	18	0.969	0.045	0.069	[0.059, 0.079]
Metric model	170.48*	23	0.968	0.047	0.062	[0.053, 0.070]
Scalar model	205.29*	28	0.962	0.051	0.061	[0.053, 0.069]
Adjusted scalar model	172.15*	24	0.968	0.047	0.060	[0.052, 0.069]
Strict model	178.99*	30	0.968	0.048	0.054	[0.047, 0.062]

Model comparison	Δ*χ*^2^ (*df*)	*p*	ΔCFI	ΔSRMR	ΔRMSEA	
*BBQ age groups*						
Metric vs. Configural	7.58 (5)	0.18	−0.001	0.001	−0.007	
Scalar vs. Metric	64.60 (5)	< 0.001	−0.008	0.005	0.002	
Adjusted Scalar vs. Metric	8.49 (4)	< 0.001	0	0.001	−0.004	
Strict vs. Adjusted Scalar	15.60 (6)	0.016	−0.001	0.002	−0.004	
*BBQ gender groups*						
Metric vs. Configural	4.86 (5)	0.43	0	−0.001	0.007	
Scalar vs. Metric	38.40 (5)	< 0.001	−0.009	0.005	0.003	
Adjusted Scalar vs. Metric	3.18 (1)	0.075	−0.001	0	−0.001	
Strict vs. Adjusted scalar	11.00 (6)	0.090	−0.002	0.003	−0.005	
*BBQ depression groups*						
Metric vs. Configural	7.60 (5)	0.18	−0.001	0.001	−0.007	
Scalar vs. Metric	61.00 (5)	< 0.001	−0.013	0.006	0.003	
Adjusted Scalar vs. Metric	3.18 (2)	0.20	0	0	−0.002	
Strict vs. Adjusted Scalar	4.72 (6)	0.058	0.001	0.001	−0.007	
*BBQ anxiety groups*						
Metric vs. Configural	7.60 (5)	0.18	−0.001	0.001	−0.007	
Scalar vs. Metric	43.60 (5)	< 0.001	−0.006	0.004	0	
Adjusted Scalar vs. Metric	2.70 (1)	0.10	0	0	−0.001	
Strict vs. Adjusted Scalar	8.00 (6)	0.24	0	0.001	−0.006	

CFI, Comparative Fit Index; SRMR, Standardized Root-Mean-Square Residual; RMSEA, Root-Mean-Square Error of Approximation; CI, Confidence Interval for RMSEA; BBQ, Brunnsviken Brief Quality of Life Inventory.

*p < 0.01.

In the scalar invariance (strong) model, configuration, loadings, and indicator means/intercepts were constrained to be equal across age groups. Fit indices were comparable to the metric model: χ^2^(28) = 204.91, *p* < 0.001, CFI = 0.964, SRMR = 0.050, RMSEA = 0.061 [90% CI: 0.053, 0.069]. However, scalar invariance was unsupported by difference tests that evaluated model similarity: Δχ^2^(5) = 64.60, *p* < 0.001; ΔCFI = −0.008. An analysis of influential parameters revealed that the indicator mean/intercept for one item-level data domain had a significant effect on the model fit: *Friends and friendship* (*p* < 0.001). Thus, an adjusted model was fitted to the data in an effort to establish partial scalar invariance: χ^2^(27) = 165.79, *p* < 0.001, CFI = 0.972, SRMR = 0.046, RMSEA = 0.055 [90% CI: 0.047, 0.063]. Partial scalar invariance across age groups was supported by non-significant difference tests that evaluated the metric and adjusted scalar model similarity: Δ*χ*^2^(4) = 8.49, *p* = 0.075; ΔCFI = 0.000.

In the residual invariance (strict) model, configuration, loadings, indicator means/intercepts (apart from the previously established noninvariant intercept), and residuals were constrained to be equal across age groups. Fit indices were comparable to the adjusted scalar model: χ^2^(33) = 178.66, *p* < 0.001, CFI = 0.970, SRMR = 0.048, RMSEA = 0.051 [90% CI: 0.044, 0.059]. However, residual invariance was unsupported by difference tests that evaluated the adjusted scalar and residual model similarity: Δ*χ*^2^(6) = 15.6, *p* = 0.016; ΔCFI = −0.001. Despite adequate fit indices in the residual model, due to the detection of partial non-invariance at the scalar level, we did not attempt to model residual invariance further.

#### 3.2.2 Measurement invariance of gender

The sample was stratified by gender (male: *n* = 568; female: *n* = 2,791). Due to the small sample size, participants identifying as non-binary (*n* = 25) were excluded from the measurement invariance (MI) analysis of MI across genders. Thereafter, BBQ item-level data were fit to one factor. The configural model, which constrained only the relative configuration of item-level data to be equal across genders, had an adequate fit to the data: *χ*^2^(18) = 141.86, *p* < 0.001, CFI = 0.974, SRMR = 0.043, RMSEA = 0.064 [90% CI: 0.054, 0.074]. A resampling approach that corrected for unbalanced groups yielded similar fit indices (CFI = 0.980, SRMR = 0.043, RMSEA = 0.054 [90% CI: 0.043, 0.064]), in turn corroborating configural invariance across gender. The metric invariance (weak) model constrained the configuration of item-level data and factor loadings to be constant across genders. Fit indices were comparable to the configural model: χ^2^(23) = 147.48, *p* < 0.001, CFI = 0.974, SRMR = 0.044, RMSEA = 0.057 [90% CI: 0.048, 0.066]. A resampling approach that corrected for unbalanced groups yielded similar fit indices (CFA = 0.980, SRMR = 0.045, RMSEA = 0.047 [90% CI: 0.037, 0.057], in turn corroborating metric invariance across gender. Moreover, metric invariance was supported by non-significant difference tests that evaluated configural and metric model similarity: Δ*χ*^2^(5) = 4.86, *p* = 0.43; ΔCFI = 0.000.

In the scalar invariance (strong) model, configuration, loadings, and indicator means/intercepts were constrained to be equal across genders. Fit indices were comparable to the metric model: χ^2^(28) = 198.01, *p* < 0.001, CFI = 0.965, SRMR = 0.049, RMSEA = 0.060 [90% CI: 0.052, 0.068]. However, scalar invariance was unsupported by difference tests that evaluated model similarity: Δχ^2^(5) = 38.40, *p* < 0.001; ΔCFI = −0.009. An analysis of influential parameters revealed that indicator means/intercepts for four item-level data domains had a significant effect on the model fit: *Friends and friendship* (*p* < 0.001), *View on life* (*p* = 0.001), *Creativity* (*p* = 0.001), and *Leisure time* (*p* = 0.007). Thus, an adjusted model was fitted to the data in an effort to establish partial scalar invariance: *χ*^2^(24) = 151.25, *p* < 0.001, CFI = 0.974, SRMR = 0.044, RMSEA = 0.056 [90% CI: 0.048, 0.065]. A resampling approach that corrected for unbalanced groups yielded similar fit indices (CFA = 0.980, SRMR = 0.046, RMSEA = 0.047 [90% CI: 0.036, 0.059], in turn corroborating partial scalar invariance across gender. Moreover, partial scalar invariance across genders was supported by non-significant difference tests that evaluated the metric and adjusted scalar model similarity: Δ*χ*^2^(1) = 3.18, *p* = 0.075; ΔCFI = −0.001.

In the residual invariance (strict) model, configuration, loadings, indicator means/intercepts (apart from previously established noninvariant intercepts), and residuals were constrained to be equal across age groups. Fit indices were comparable to the adjusted scalar model: *χ*^2^(30) = 164.65, *p* < 0.001, CFI = 0.972, SRMR = 0.047, RMSEA = 0.052 [90% CI: 0.044, 0.059]. A resampling approach that corrected for unbalanced groups yielded similar fit indices (CFA = 0.975, SRMR = 0.052, RMSEA = 0.046 [90% CI: 0.036, 0.055], in turn corroborating residual invariance across gender. Moreover, residual invariance across genders was supported by difference tests that evaluated the adjusted scalar and residual model similarity: Δχ^2^(6) = 11.00, *p* = 0.090; ΔCFI = −0.002.

#### 3.2.3 Measurement invariance of clinical–subclinical levels of depression

Participants were split into groups according to their scores on the PHQ-9, representing above (*n* = 2,353) and below (*n* = 1,031) threshold for clinical depression (i.e., PHQ-9 ≥ 10). Thereafter, BBQ item-level data were fit to one factor. The configural model, which constrained only the relative configuration of item-level data to be equal across groups, had an adequate fit to the data: χ^2^(18) = 165.78, *p* < 0.001, CFI = 0.962, SRMR = 0.046, RMSEA = 0.070 [90% CI: 0.060, 0.080]. A resampling approach that corrected for unbalanced groups yielded similar fit indices (CFA = 0.955, SRMR = 0.048, RMSEA = 0.072 [90% CI: 0.069, 0.076], in turn corroborating configural invariance across clinical–subclinical levels of depression. The metric invariance (weak) model, which constrained the configuration of item-level data and factor loadings to be constant across groups had fit indices that were comparable to the configural model: χ^2^(23) = 175.97, *p* < 0.001, CFI = 0.961, SRMR = 0.048, RMSEA = 0.063 [90% CI: 0.054, 0.072]. A resampling approach that corrected for unbalanced groups yielded similar fit indices (CFA = 0.956, SRMR = 0.048, RMSEA = 0.072 [90% CI: 0.069, 0.075], in turn corroborating metric invariance across clinical–subclinical levels of depression. Moreover, metric invariance was supported by non-significant difference tests that evaluated configural and metric model similarity: Δχ^2^(5) = 7.60, *p* = 0.18; ΔCFI = −0.001.

In the scalar invariance (strong) model, configuration, loadings, and indicator means/intercepts were constrained to be equal across clinical–subclinical groups of depression. Fit indices were comparable, albeit slightly worse, to the metric model: *χ*^2^(28) = 232.21, *p* < 0.001, CFI = 0.948, SRMR = 0.054, RMSEA = 0.066 [90% CI: 0.058, 0.074]. However, scalar invariance was unsupported by difference tests that evaluated model similarity: Δχ^2^(5) = 61.00, *p* < 0.001; ΔCFI = −0.013. An analysis of influential parameters revealed that indicator means/intercepts for three item-level data domains had a significant effect on the model fit: C*reativity* (*p* < 0.001), *Friends and friendship* (*p* = 0.002), and *View of self* (*p* < 0.001). Thus, an adjusted model was fitted to the data in an effort to establish partial scalar invariance: *χ*^2^(25) = 178.60, *p* < 0.001, CFI = 0.961, SRMR = 0.048, RMSEA = 0.060 [90% CI: 0.052, 0.069]. A resampling approach that corrected for unbalanced groups yielded similar fit indices (CFA = 0.955, SRMR = 0.048, RMSEA = 0.072 [90% CI: 0.069, 0.075], in turn corroborating partial scalar invariance across clinical–subclinical levels of depression. Moreover, partial scalar invariance across clinical–subclinical groups of depression was supported by non-significant difference tests that evaluated the metric and adjusted scalar model similarity: Δ*χ*^2^(2) = 3.18, *p* = 0.20; ΔCFI = 0.000.

In the residual invariance (strict) model, configuration, loadings, indicator means/intercepts (apart from previously established noninvariant intercepts), and residuals were constrained to be equal across clinical–subclinical groups of depression. Fit indices were comparable to the adjusted scalar model: *χ*^2^(31) = 182.32, *p* < 0.001, CFI = 0.962, SRMR = 0.049, RMSEA = 0.054 [90% CI: 0.046, 0.061]. Moreover, a resampling approach that corrected for unbalanced groups yielded similar fit indices (CFA = 0.956, SRMR = 0.048, RMSEA = 0.072 [90% CI: 0.069, 0.075], in turn indicating residual invariance across clinical–subclinical levels of depression. However, residual invariance across clinical–subclinical groups was supported by non-significant difference tests that evaluated the adjusted scalar and residual model similarity: Δ*χ*^2^(6) = 4.72, *p* = 0.058; ΔCFI = 0.001.

#### 3.2.4 Measurement invariance of clinical–subclinical levels of anxiety

Participants were split into groups according to their scores on the GAD-7, representing above (*n* = 2,202) and below (*n* = 1,182) threshold for clinical anxiety (i.e., GAD-7 ≥ 8). Thereafter, BBQ item-level data were fit to one factor. The configural model, which constrained only the relative configuration of item-level data to be equal across groups, had an adequate fit to the data: *χ*^2^(18) = 161.25, *p* < 0.001, CFI = 0.969, SRMR = 0.045, RMSEA = 0.069 [90% CI: 0.059, 0.079]. A resampling approach that corrected for unbalanced groups yielded similar fit indices (CFA = 0.966, SRMR = 0.043, RMSEA = 0.069 [90% CI: 0.066, 0.073], in turn corroborating configural invariance across clinical–subclinical levels of anxiety. The metric invariance (weak) model, which constrained the configuration of item-level data and factor loadings to be constant across groups had fit indices that were comparable to the configural model: *χ*^2^(23) = 170.48, *p* < 0.001, CFI = 0.962, SRMR = 0.051, RMSEA = 0.061 [90% CI: 0.053, 0.069]. A resampling approach that corrected for unbalanced groups yielded similar fit indices (CFA = 0.966, SRMR = 0.045, RMSEA = 0.070 [90% CI: 0.066, 0.074], in turn indicating metric invariance across clinical–subclinical levels of anxiety. Moreover, metric invariance was supported by non-significant difference tests that evaluated model configural and metric similarity: Δ*χ*^2^(5) = 7.60, *p* = 0.18; ΔCFI = −0.001.

In the scalar invariance (strong) model, configuration, loadings, and indicator means/intercepts were constrained to be equal across clinical–subclinical groups of anxiety. Fit indices were comparable to the metric model: *χ*^2^(28) = 205.29, *p* < 0.001, CFI = 0.962, SRMR = 0.047, RMSEA = 0.061 [90% CI: 0.053, 0.069]. However, scalar invariance was unsupported by difference tests that evaluated model similarity: Δ*χ*^2^(5) = 43.60, *p* < 0.001; ΔCFI = −0.006. An analysis of influential parameters revealed that indicator means/intercepts for three item-level data domains had a significant effect on the model fit: *Leisure time* (*p* = 0.010), *View on life* (*p* = 0.010), *Friends and friendship* (*p* < 0.001), and *View of self* (*p* = 0.010). Thus, an adjusted model was fitted to the data in an effort to establish partial scalar invariance: χ^2^(24) = 172.15, *p* < 0.001, CFI = 0.968, SRMR = 0.047, RMSEA = 0.060 [90% CI: 0.052, 0.069]. A resampling approach that corrected for unbalanced groups yielded similar fit indices (CFA = 0.966, SRMR = 0.045, RMSEA = 0.070 [90% CI: 0.067, 0.072], in turn indicating partial scalar invariance across clinical–subclinical levels of anxiety. Moreover, partial scalar invariance across clinical–subclinical groups of anxiety was supported by non-significant difference tests that evaluated the metric and adjusted scalar model similarity: Δχ^2^(1) = 2.70, *p* = 0.10; ΔCFI = 0.000.

In the residual invariance (strict) model, configuration, loadings, indicator means/intercepts (apart from previously established noninvariant intercepts), and residuals were constrained to be equal across clinical–subclinical groups of anxiety. Fit indices were comparable to the adjusted scalar model: χ^2^(30) = 178.99, *p* < 0.001, CFI = 0.968, SRMR = 0.048, RMSEA = 0.054 [90% CI: 0.047, 0.062]. A resampling approach that corrected for unbalanced groups yielded similar fit indices (CFA = 0.966, SRMR = 0.045, RMSEA = 0.070 [90% CI: 0.066, 0.074], in turn indicating residual invariance across clinical–subclinical levels of anxiety. Moreover, residual invariance across clinical–subclinical groups was supported by difference tests that evaluated the adjusted scalar and residual model similarity: Δ*χ*^2^(6) = 8.00, *p* = 0.24; ΔCFI = 0.000.

### 3.3 Preliminary analysis of BBQ cutoff points

The distribution of BBQ scores across groups of clinical–subclinical depression and anxiety, along with significance tests between groups are presented in the Supplementary Material (see Tables S1,S2).

#### 3.3.1 Receiver operating characteristic curve analysis

**Figure 2:**
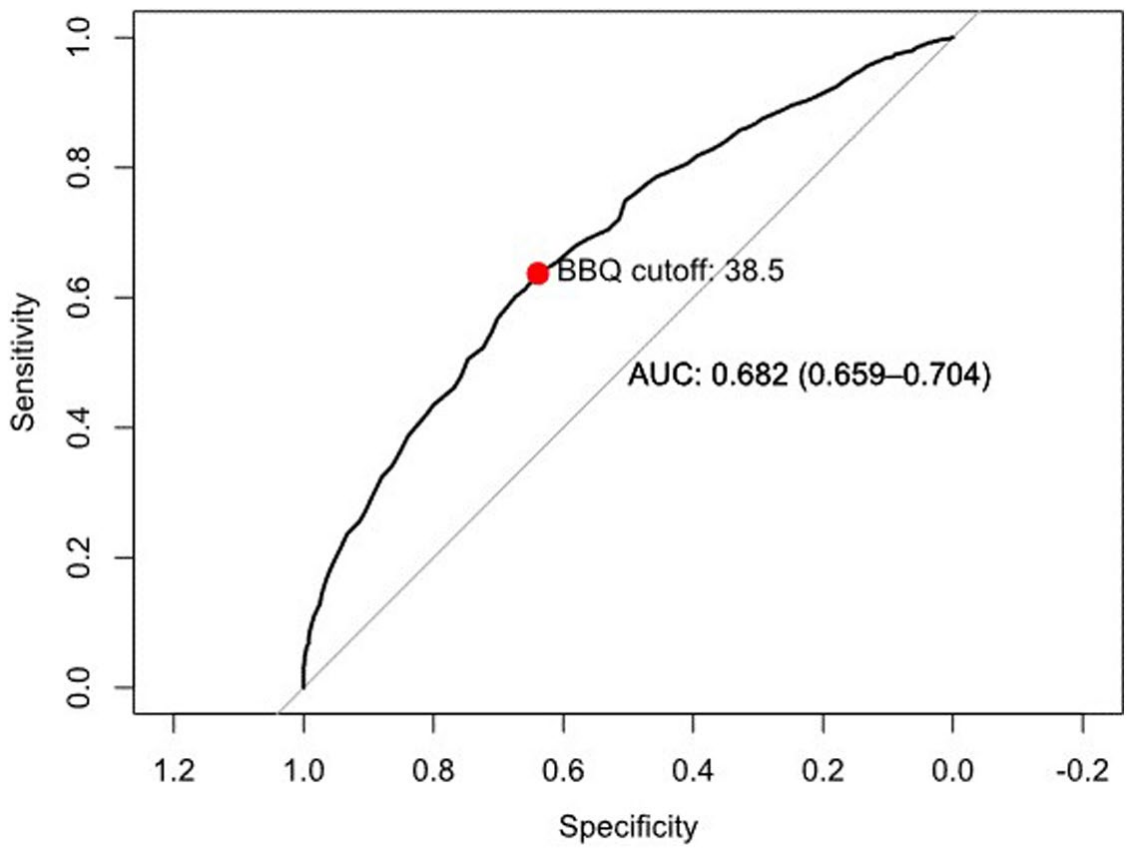
Receiver operating characteristic (ROC) curve for assessing the discriminative ABILITY of the BBQ scale based on PHQ-9 and GAD-7 criteria.

The ROC curve revealed that the BBQ scale displayed moderate discriminative ability (see Figure 2). The Area Under the Curve (AUC) was found to be 0.682, which suggests that the BBQ has an adequate capability in distinguishing between individuals with and without symptoms of clinical depression or anxiety.

The ROC curve analysis identified an optimal cutoff point of 38.5 for the BBQ scale. This point was determined as the threshold that maximizes both sensitivity and specificity, thereby enhancing its ability to reliably differentiate between varying levels of QoL in a treatment-seeking population.

## 4 Discussion

The BBQ is a brief, freely available measure of subjective QoL. The current study corroborates previous findings that have shown the BBQ to be a reliable and valid measure of QoL (Lindner et al., 2016; Biliunaite et al., 2021; Pantić et al., 2023). Our aim was to assess whether the factor structure of the BBQ remained stable across different age groups, genders, depressive symptom severity, and anxiety symptom severity (i.e., assess measurement invariance [MI]), and to identify potential cutoff points for the BBQ. The BBQ demonstrated MI at the metric level and partial MI at the scalar level across different age groups, genders, depressive symptom severity groups, and anxiety symptom severity groups. Moreover, partial MI at the strict-residual level remained stable for all groups except for genders. With regards for potential cutoff points, the results indicated that a BBQ total score below 39 is associated with greater psychopathology; determining exact cut off scores would however require other study designs and measures, which should be the topic of future research.

Across age groups, the BBQ demonstrated partial scalar invariance when indicator means/intercepts from the domain *Friends and friendship* was estimated separately, which then remained stable at the strict-residual level. Across genders, the BBQ demonstrated partial scalar invariance when indicator means/intercepts from the domains *Friends and friendship*, *View on life*, *Creativity*, and *Leisure time* were estimated separately, which did not remain stable at the strict-residual level. Across depression groups, the BBQ demonstrated partial scalar invariance when indicator means/intercepts from the domains C*reativity*, *Friends and friendship*, and *View of self* were estimated separately, which then remained stable at the strict-residual level. Finally, across anxiety groups, the BBQ demonstrated partial scalar invariance when indicator means/intercepts from the domains *Leisure time*, *View on life*, *Friends and friendship*, and *View of self* were estimated separately, which then remained stable at the strict-residual level. Moreover, these results remained stable when a resampling approach that corrects for unbalanced groups was implemented (Yoon and Lai, 2018). Taken together, the results indicate that the BBQ can be used as a reliable assessment of QoL across divergent groups.

This study has limitations. Age groups were constructed based on median age. This may have limited the analysis as it does not consider possible more complex associations between age and QoL, and thus risks overfitting to our specific study population. Nonetheless, a median split does ensure that groups are statistically equivalent in size. Another limitation relates to our decision to exclude participants with non-binary gender identities from the analysis. Although this was done to ensure that comparisons between groups are fair and representative, valuable information may have been lost due to this decision. Another limitation relates to the absence of clinical interviews to confirm our categorization of individuals into depression/non-depression and anxiety/non-anxiety groups (Carlbring et al., 2002). Instead, scores on the PHQ-9 and GAD-7 that are commonly considered good diagnostic indicators of depression and anxiety were used to categorize people with clinical and subclinical depression and anxiety. However, both the PHQ-9 and GAD-7 (Johnson et al., 2019; Byrd-Bredbenner et al., 2021; Martin-Key et al., 2022) reliably indicate depressive and anxiety disorders. Moreover, all participants were treatment-seeking which, in turn, substantiates our categorization. Another limitation is the absence of cross-validation for the proposed cut-off point, along with a lack of longitudinal data to assess how QoL scores may change over time, the latter not allowing us to examine longitudinal measurement invariance. Finally, the present study is limited to inferring only about scores on the lower end of the spectrum of BBQ total scores. By only analyzing BBQ total scores with regard for which total scores have the highest probability of being associated with psychopathological symptom presentation, the upper end of the BBQ total score spectrum remains unevaluated. The absence of a psychological disorder is not equal to the presence of well-being, and thus future studies must establish cutoff values for the upper spectrum of the BBQ total using previously validated measures of QoL or wellbeing.

The present study has numerous strengths. Principally, leveraging data from both included and excluded participants the treatment study, this analysis does not suffer from a restriction of range. Other strengths include the exclusive inclusion of treatment-seeking individuals and a large sample size.

## 5 Implications and conclusions

This study established that the BBQ is invariant to measurement across different groups, such as age, gender, and varying levels of psychopathology. Investigations of the scales used in healthcare and research are essential to ensure their reliability for different groups (e.g., vulnerable persons), as well as to ensure health equity and general fairness. Moreover, indicators of individual subjective QoL can provide additive information about treatment efficacy that cannot be captured through common symptom-focused measurements. The BBQ has consistently been demonstrated to be a reliable and valid measurement of QoL. This study adds to the existing literature by establishing at least weak-metric MI and partial scalar MI across all groups, as well as partial strict-residual MI for age groups and clinical–subclinical depression and anxiety groups. Finally, the present study suggests a cutoff point for the BBQ that is clinically relevant.

## Data availability statement

The raw data supporting the conclusions of this article will be made available by the authors, without undue reservation.

## Ethics statement

The studies involving humans were approved by Etikprövningsmyndigheten Box 2,110 750 02 Uppsala SWEDEN. The studies were conducted in accordance with the local legislation and institutional requirements. The participants provided their written informed consent to participate in this study.

## Author contributions

JIH: Writing – original draft, Formal analysis, Methodology, Data curation, Investigation, Validation, Visualization, Writing – review & editing. PL: Writing – review & editing, Conceptualization, Resources, Methodology, Supervision. BB: Writing – original draft, Investigation, Methodology, Validation. PC: Writing – review & editing, Data curation, Conceptualization, Resources, Project administration, Validation, Supervision.
