# Supplementary material for: Exploring cutoff points and measurement invariance of the Brunnsviken brief quality of life inventory
Source: Front Psychol. 2024 Jan 8;14:1305682. doi: 10.3389/fpsyg.2023.1305682 (PMC10801901; doi:10.3389/fpsyg.2023.1305682)
Supplement: Supplementary file 1 [file Data_Sheet_1.docx]

Supplementary Material

# Supplementary Figures and Tables

| **Table S1.**  Descriptive statistics of BBQ item-level data and total scores by depression and anxiety groups. | | | | |
| --- | --- | --- | --- | --- |
|  |  |  | Range | |
|  | Mean | SD | Min | Max |
| **Clinical depression** (*n* = 2369) |  |  |  |  |
| BBQ total score | 32.13 | 16.88 | [0, 92] | |
| Leisure time | 5.06 | 4.13 | [0, 16] | |
| View on life | 5.71 | 4.46 | [0, 16] | |
| Creativity | 5.35 | 4.52 | [0, 16] | |
| Learning | 5.71 | 4.47 | [0, 16] | |
| Friends and friendship | 5.61 | 4.95 | [0, 16] | |
| View of Self | 4.70 | 3.99 | [0, 16] | |
| **Subclinical depression** (*n* = 1032) |  |  |  |  |
| BBQ total score | 45.16 | 17.64 | [0, 96] | |
| Leisure time | 7.52 | 4.15 | [0, 16] | |
| View on life | 8.26 | 4.64 | [0, 16] | |
| Creativity | 6.94 | 4.55 | [0, 16] | |
| Learning | 7.98 | 4.71 | [0, 16] | |
| Friends and friendship | 6.87 | 4.97 | [0, 16] | |
| View of Self | 7.58 | 4.09 | [0, 16] | |
| **Clinical anxiety** (*n* = 2217) |  |  |  |  |
| BBQ total score | 33.82 | 17.63 | [0, 92] | |
| Leisure time | 5.28 | 4.17 | [0, 16] | |
| View on life | 6.16 | 4.59 | [0, 16] | |
| Creativity | 5.49 | 4.60 | [0, 16] | |
| Learning | 5.95 | 4.55 | [0, 16] | |
| Friends and friendship | 5.90 | 5.02 | [0, 16] | |
| View of Self | 5.04 | 4.17 | [0, 16] | |
| **Subclinical anxiety** (*n* = 1184) |  |  |  |  |
| BBQ total score | 40.32 | 18.30 | [0, 96] | |
| Leisure time | 6.78 | 4.33 | [0, 16] | |
| View on life | 7.09 | 4.76 | [0, 16] | |
| Creativity | 6.46 | 4.51 | [0, 16] | |
| Learning | 7.26 | 4.75 | [0, 16] | |
| Friends and friendship | 6.15 | 4.93 | [0, 16] | |
| View of Self | 6.57 | 4.18 | [0, 16] | |

Participants with scores on the PHQ-9 indicative of clinical depression (i.e., PHQ-9 ≥ 10) had on average 13.03-point lower total scores on the BBQ [95% CI: -14.30, -11.76], compared with participants with scores indicative of subclinical depression [*t*(1887) = -20, *p* < .001]. Similarly, participants with scores on the GAD-7 indicative of clinical anxiety (i.e., GAD-7 ≥ 8) had on average 6.50-point lower total scores on the BBQ [95% CI: -7.78, -5.23], compared with participants with scores indicative of subclinical anxiety [*t*(2340) = -10, *p* < .001].

In order to attain a more comprehensive understanding of the impact of depression and anxiety on QoL, the sample was categorized into distinct groups based on concurrent stratification for both depression and anxiety (see Table S2). Participants with scores on the PHQ-9 and GAD-7 indicative of cooccurring clinical depression and clinical anxiety had, on average, the lowest scores on the BBQ (Mean = 31.52), while participants with scores on the PHQ-9 and GAD-7 indicative of neither subclinical depression nor subclinical anxiety had, on average, the highest scores on the BBQ (Mean = 45.55). However, comparing the difference in total scores on the BBQ between participants with scores on the PHQ-9 and GAD-7 indicative of neither subclinical depression nor subclinical anxiety (Mean = 45.55) and scores indicative of clinical depression with subclinical anxiety (Mean = 44.53) was non-significant [*t*(838) = 0.91, *p* = .40).

| **Table S2.**  Descriptive statistics for BBQ total scores by combined depression and anxiety groups. | | | | |
| --- | --- | --- | --- | --- |
|  |  |  | Range | |
|  | Mean | SD | Min | Max |
| **Clinical depression & clinical anxiety** (*n* = 1825) |  |  |  |  |
| BBQ total score | 31.52 | 16.81 | [0, 92] | |
| **Subclinical depression & clinical anxiety** (*n* = 392) |  |  |  |  |
| BBQ total score | 44.53 | 17.45 | [0, 92] | |
| **Clinical depression & subclinical anxiety** (*n* = 544) |  |  |  |  |
| BBQ total score | 34.16 | 16.98 | [0, 92] | |
| **Subclinical depression & subclinical anxiety** (*n* = 640) |  |  |  |  |
| BBQ total score | 45.55 | 17.76 | [6, 92] | |
